# Development and Optimization of Grape Skin Extract-Loaded Gelatin–Alginate Hydrogels: Assessment of Antioxidant and Antimicrobial Properties

**DOI:** 10.3390/pharmaceutics17060790

**Published:** 2025-06-17

**Authors:** Jovana Bradic, Anica Petrovic, Aleksandar Kocovic, Vukasin Ugrinovic, Suzana Popovic, Andrija Ciric, Zoran Markovic, Edina Avdovic

**Affiliations:** 1 Department of Pharmacy, Faculty of Medical Sciences, University of Kragujevac, Svetozara Markovica, 69, 34000 Kragujevac, Serbia; jovanabradickg@gmail.com (J.B.); petkovicanica0@gmail.com (A.P.); 2 Center of Excellence for Redox Balance Research in Cardiovascular and Metabolic Disorders, Zmaj Jovina 30, 34000 Kragujevac, Serbia; 3 Innovation Center of the Faculty of Technology and Metallurgy Ltd., Karnegijeva 4, 11120 Belgrade, Serbia; 4 Centre for Molecular Medicine and Stem Cell Research, Faculty of Medical Sciences, University of Kragujevac, Svetozara Markovica, 69, 34000 Kragujevac, Serbia; popovic007@yahoo.com; 5 Faculty of Science, University of Kragujevac, Radoja Domanovica 12, 34000 Kragujevac, Serbia; andrija.ciric@pmf.kg.ac.rs; 6 Department of Natural Sciences and Mathematics, State University of Novi Pazar, Vuka Karadzica bb, 36300 Novi Pazar, Serbia; zmarkovic@uni.kg.ac.rs; 7 Institute for Information Technologies, University of Kragujevac, Jovana Cvijica bb, 34000 Kragujevac, Serbia; edina.avdovic@pmf.kg.ac.rs

**Keywords:** grape skins, hydrogels, biopolymers, antioxidant, antimicrobial activity

## Abstract

**Background:** In this study, we aimed to develop and optimize unique eco-friendly gelatin–alginate hydrogels enriched with sustainable grape skin extract for advanced wound healing applications. **Methods:** Following confirmation of the extract’s antioxidant activity, hydrogels were synthesized by varying gelatin content and CaCl_2_ concentration to achieve desirable crosslinking density, mechanical properties, and extract release behavior. Physicochemical characterization of hydrogels included equilibrium swelling analysis, mechanical testing, FTIR analysis, and in vitro release of extract from hydrogel. Moreover, the biocompatibility of hydrogels enriched with extract was assessed via MTT assay, while antimicrobial activity was tested against *Staphylococcus aureus* ATCC 25923, *Escherichia coli* ATCC 25922, *Pseudomonas aeruginosa* ATCC 10145, and *Candida albicans* ATCC 10231. The antioxidant capacity of the hydrogels was evaluated using DPPH, ABTS, and FRAP assays. **Results:** Our results showed that higher gelatin and CaCl_2_ concentrations produced denser crosslinked networks, leading to reduced swelling and increased stiffness. Additionally, the extract exhibited a biphasic release profile from hydrogels, featuring an initial rapid release followed by sustained release over 24 h. **Conclusions:** The hydrogels maintained high biocompatibility and demonstrated selective antimicrobial activity, particularly against *Escherichia coli*, and satisfactory antioxidant activity. Obtained multifunctional sustainable hydrogels enriched with grape skin extract represent promising agents for managing skin conditions associated with oxidative stress and bacterial infections.

## 1. Introduction

The management of chronic and acute wounds remains a significant clinical challenge, necessitating the development of advanced biomaterials that surpass the limitations of conventional dressings. Hydrogels, characterized by their three-dimensional, hydrophilic polymeric networks, have emerged as superior candidates in wound care due to their intrinsic properties, such as a high level of water retention, biocompatibility, and capacity to maintain a moist microenvironment conducive to cellular proliferation and tissue regeneration. These features facilitate accelerated wound healing by promoting re-epithelialization, reducing microbial colonization, and enabling controlled therapeutic delivery [1,2]. Despite these advantages, the engineering of hydrogels with tailored mechanical integrity, degradation kinetics, and precise drug release profiles remains an ongoing challenge, particularly for complex wound environments requiring multifunctional therapeutic interventions [3].

The majority of wound healing hydrogels are formulated from synthetic polymers, which may produce toxic degradation products and pose environmental concerns, underscoring the need for natural, bioactive alternatives that can provide enhanced biocompatibility and therapeutic benefits [4]. Among natural polymers, gelatin and alginate have garnered considerable attention for hydrogel fabrication owing to their complementary physicochemical and biological properties. Alginate’s polyanionic structure confers exceptional exudate absorption and gelation capabilities, fostering a moist wound milieu that accelerates tissue repair [5,6,7]. However, alginate hydrogels are often hindered by rapid degradation and elevated permeability, which compromise their efficacy in sustained drug delivery and wound barrier functions. Gelatin incorporation addresses these limitations by enhancing mechanical robustness and modulating biodegradation rates, resulting in hybrid gelatin–alginate hydrogels with improved stability and functional longevity [8]. The synergistic interplay between these biopolymers renders them highly suitable for diverse biomedical applications, including drug delivery systems, tissue scaffolding, and wound dressings. Nonetheless, optimizing polymer concentration, cross-linking strategies, and environmental parameters is critical to achieving hydrogels with desirable swelling behavior, mechanical strength, and controlled bioactive release tailored to specific clinical needs [9,10].

The healing effectiveness of gelatin–alginate hydrogels can be enhanced by incorporating bioactive natural substances that possess antioxidant and antimicrobial effects [1]. While gelatin–alginate systems have previously shown antimicrobial potential, especially when combined with antibiotics like tetracycline or ciprofloxacin, their use with natural components, such as grape pomace, remains underexplored [5,11,12]. Recent phytochemical investigations have highlighted grape skin extracts as rich reservoirs of polyphenolic constituents, such as anthocyanins, flavonoids, and resveratrol, which possess potent free radical scavenging, anti-inflammatory, and antimicrobial activities. Although grape skin extract is rich in bioactive compounds with promising therapeutic potential, its application in advanced pharmaceutical formulations remains limited. Commercial formulations are mostly restricted to cosmeceuticals with minimal clinical validation [13,14,15]. In this context, the incorporation of grape skin extract into hydrogel matrices offers a promising avenue to develop multifunctional wound dressings that not only provide structural support but also actively modulate the wound microenvironment to mitigate oxidative stress and microbial infection.

In light of these considerations, the present study aims to optimize the fabrication parameters of gelatin–alginate hydrogels loaded with eco-friendly grape skin extract and to systematically evaluate their antioxidant and antimicrobial properties. The novelty of this work lies in the strategic combination of biopolymers with a natural, bioactive extract to enhance the functional properties of hydrogels, offering a sustainable and efficacious platform for applications in wound healing, drug delivery, and tissue engineering.

## 2. Materials and Methods

### 2.1. Grape Skin Extract Preparation

Grape skin samples for extraction were obtained from a local winery located in the central part of Serbia, Šumadija. These skins were a by-product of rosé wine production using the Cabernet Sauvignon grape type. Before extraction, the samples were subjected to a meticulous preparation process that included thorough washing and drying. Ultrasound-assisted extraction (UAE) is an environmentally friendly method that was used in this study to extract bioactive compounds from grape skins. Each 20 g sample of dried grape skins was submerged in 200 mL of ethyl acetate. The extraction was conducted in an ultrasonic bath operating at a frequency of 40 kHz and a power output of 150 W, with the process lasting 30 min at a controlled temperature of 40 °C. Following extraction, the mixture was centrifuged at 2490× *g*-force for 10 min to separate solid residues effectively. The filtrates were concentrated using a rotary evaporator, with the temperatures kept below 40 °C to preserve sensitive bioactive compounds. The concentrated extracts were stored at 5 °C [16,17,18].

### 2.2. Determination of Total Phenolic Content in Grape Skin Extract

The total phenolic content (TPC) of grape skin extracts was determined using a modified version of the previously reported Folin–Ciocalteu method [16,19,20,21]. Briefly, 50 µL of the extract solution (tested at different dilutions to ensure the absorbance values remained within the linear range of the assay) and 250 µL of the Folin–Ciocalteu reagent (Sigma-Aldrich, St. Louis, MO, USA) (diluted 1:2 with distilled water) were mixed in a volumetric flask of 5 mL. The mixture was allowed to react for five minutes at room temperature. Subsequently, 750 µL of 20% sodium carbonate (Na_2_CO_3_) solution was added, and the final volume (5 mL) was adjusted with deionised water. The samples were then allowed to stand at room temperature in the dark for ninety minutes. Post-incubation, absorbance at 765 nm was measured using a UV-Vis spectrophotometer. The TPC was expressed as milligrams of gallic acid equivalents (GAE) per gram of dry grape skin extract (E) (mg GAE/g E). All measurements were performed in triplicate to ensure reliability [22].

### 2.3. In Vitro Antioxidant Potential Assessment of Grape Skin Extract

The antioxidant capacity of grape skin extracts was evaluated through three well-established in vitro assays: DPPH, ABTS, and FRAP. These assays are widely recognized for their high reproducibility and reliability in determining the antioxidant potential across diverse samples. All chemicals for this part of the experiment were purchased from Sigma-Aldrich (St. Louis, MO, USA).

#### 2.3.1. DPPH Radical Scavenging Assay

The DPPH assay was employed to evaluate the free radical scavenging activity of grape skin extract. A series of different concentrations of the corresponding extract in methanol was prepared. An equal volume of the DPPH• radical solution was added to each sample solution in cuvettes, and the mixture was then incubated for 30 min at 25 °C. DPPH• free control samples were used to minimize any potential interference. The half-maximal effective scavenging concentration (SC_50_) was determined for each sample. Quercetin, ascorbic acid, and nordihydroguaiaretic acid (NDGA) served as positive controls to validate the assay’s reliability [16,23,24,25].

#### 2.3.2. ABTS Radical Cation Decolorization Assay

The ABTS assay, another robust method for evaluating radical scavenging capacity, was applied to analyze the extract. Methanol was used as a solvent for the preparation of different concentrations of the examined extract. The ABTS radical cation (ABTS^•+^) was generated according to a known procedure [12]. To achieve an absorbance of roughly 0.70 at a wavelength of 734 nm, the ABTS^•+^ solution was diluted with methanol during the experiment [16,23,24].

#### 2.3.3. Ferric Reducing Antioxidant Power Assay

The Ferric Reducing Antioxidant Power (FRAP) assay was utilized to assess the reducing capacity of grape skin extracts. First, stock solutions were made in methanol, and then phosphate buffer with a pH of 7.4 was used to dilute them appropriately. The prepared samples were incubated at room temperature for 10 min before their absorbance was measured at 700 nm. Ascorbic acid, prepared at a concentration of 5 µg/mL, served as the reference compound in this investigation [16,23].

### 2.4. HPLC Analysis of Grape Skin Extract

The identification and quantification of particular phenolic compounds in grape skin extracts were conducted utilizing high-performance liquid chromatography (HPLC). The analyses were conducted utilizing a Shimadzu Prominence HPLC system (Kyoto, Japan) that is fitted with a photodiode array (PDA) detector (SPD-M20A). Separation was achieved using a Hypersil Gold aQ C18 column (Thermo Scientific, Waltham, MA, USA; 150 × 4.6 mm, 5 µm particle size), maintained at ambient temperature. The mobile phase was composed of two solvents: (A) 0.1% formic acid in deionized water and (B) 0.1% formic acid in acetonitrile. A gradient elution program was utilized, characterized by the subsequent profile: Initially, 2% B was applied for a duration of 0 to 2 min, subsequently increasing to 95% B over a period of 45 min. The flow rate was set at 1 mL/min, and the injection volume was 20 µL. UV detection was conducted at various wavelengths (for instance, 280 nm for phenolic acids and flavonoids) to encompass a wide spectrum of phenolic compounds. The processing of data was conducted utilizing Shimadzu LabSolutions software (Kyoto, Japan; Software version 6.115), and the identification of phenolic compounds was accomplished through the comparison of retention times and UV spectra with established standards [16,19,20,21,25]. All experiments were conducted in triplicate to ensure reproducibility and precision.

### 2.5. Synthesis of Hydrogels

Hydrogels were prepared using the modified mold casting technique from previous research [5,26]. Alginate and gelatin were measured in different ratios (Table 1) and added to either 10 mL of purified water or a solution containing 8 mg/mL of extract dissolved in purified water. The preparations were mixed using a mechanical stirrer at 500 rpm until homogeneous mixtures were obtained. The mixtures were then transferred to an ultrasound bath for 15 min and left to degas for 2 h. The mixtures were then poured into molds of desired dimensions. The molds were transferred to the freezer, where they remained for 2 h at −20 °C. The hydrogels were then taken out of the freezer and immediately immersed in a 0.5 M or 1 M CaCl_2_ solution for 5 min and were then left to dry at room temperature for several hours before further use. The optimal concentration of grape skin extract for incorporation into the hydrogels was selected based on preliminary solubility and stability assessments, alongside guidance from the relevant literature demonstrating effective topical concentrations of grape pomace extracts [27,28]. Moreover, gelatin and alginate ratios were partly based on previous studies; however, they were adjusted to suit the specific requirements of our formulation intended for cutaneous application for wound healing [5,29].

### 2.6. Hydrogel Characterization

The obtained hydrogels without grape skin extract underwent several characterization techniques, including equilibrium swelling degree, scanning electron microscopy, mechanical properties evaluation, and FTIR analysis. On the other hand, hydrogels enriched with grape skin extract were subjected to in vitro extract release study and analysis of the kinetics of drug release.

#### 2.6.1. Equilibrium Swelling Degree

The equilibrium swelling degree (*ESD*) of the hydrogels was measured after immersion in phosphate buffer saline (PBS) of pH = 7.4. The samples were incubated at 37 °C for 24 h to ensure equilibrium swelling. The water content was calculated using the following equation [30]:

$$ESD\left(\%\right)=\frac{m_{eq}-m_{0}}{m_{0}}\times 100$$

where *m_eq_* represents the mass of the fully swollen hydrogel at equilibrium, and *m*_0_ denotes the dry weight of the xerogel.

#### 2.6.2. Scanning Electron Microscopy

The morphology of the hydrogels was examined using a field emission scanning electron microscope (FE-SEM; Tescan MIRA 3 XMU, Brno, Czech Republic) operated at 20 kV. Prior to imaging, all samples were swollen to equilibrium in PBS, frozen at −20 °C overnight, and subsequently lyophilized. Freeze-drying was performed using a Beta 2–8 LD plus freeze-dryer (Martin Christ GmbH, Osterode am Harz, Germany) under the following conditions: −60 °C at 0.011 mbar for 24 h, followed by −75 °C at 0.0012 mbar for 1 h to ensure complete removal of residual moisture. The dried samples were then sputter-coated with a thin layer of gold using a POLARON SC502 sputter coater (Essex Junction, VT, USA) to minimize electrostatic charging during SEM analysis.

#### 2.6.3. Mechanical Properties Evaluation

The mechanical properties of the hydrogels were assessed using a Universal Testing Machine AG-Xplus (Shimadzu, Kyoto, Japan) equipped with a 1000 N load cell (operational range: 0.01 to 1000 N). All tests were conducted on hydrogels after achieving ESD in PBS. Unconfined compression tests were conducted on cylindrical specimens (8 mm height, 5 mm diameter) at a compression rate of 6 mm/min, up to 100% strain. The contact between the compression plate and the hydrogel was detected automatically by setting a contact force threshold of 0.1 N. Compressive strength was determined at the point of failure, and the compressive modulus was calculated as the slope of the linear portion of the stress-strain curve (0–10% strain range). Each hydrogel type was tested using at least three specimens, with mean values and standard deviations reported [30].

#### 2.6.4. FTIR Analysis

Fourier transform infrared spectroscopy (FTIR) was employed to identify the chemical composition and functional groups of the hydrogel samples. The spectra were recorded in absorbance mode using a Nicolet™ iS™10 FT-IR Spectrometer (Thermo Fisher Scientific, Madison, WI, USA), equipped with Smart iTR™ Attenuated Total Reflectance (ATR) accessories. Data collection was conducted over the range of 400–4000 cm^−1^, with a resolution of 4 cm^−1^ and an average of 20 scans per sample [30].

#### 2.6.5. In Vitro Extract Release Study

Gelatin/alginate hydrogels, incorporating grape skin extract, were molded into circular discs (8 mm in diameter, 5 mm in thickness). For the in vitro release study, the hydrogels were immersed in 40 mL of PBS solution (pH 7.4) and incubated at 37 °C in a thermostatic incubator. At predetermined time intervals, 2 mL aliquots of the release medium were collected and analyzed using a Shimadzu UV-1800 UV/V is spectrophotometer (Kyoto, Japan) at 280 nm. Grape skin extract release was quantified, and cumulative release was determined using a standard calibration curve.

#### 2.6.6. Analysis of the Kinetics of Drug Release

The release kinetics of extract from Alg/Gel hydrogels were analyzed by applying the Korsmeyer–Peppas mathematical model $\alpha =k_{p}t^{n}$, where α represents the fraction of released drug at time t, k_p_ is the Korsmeyer–Peppas constant, and n is the diffusional exponent, indicative of the release mechanism [31,32]. The model was applied to the initial portion of the release data (up to ~60% cumulative release), which is considered most appropriate for this approach.

### 2.7. In Vitro Biocompatibility Testing of Hydrogels Based on Sustainable Grape Skin Extract

In vitro biocompatibility assessment of hydrogels was conducted by evaluating their cytotoxicity through extract and direct contact tests using the MTT assay. Additionally, cell morphology was analyzed via microscopy, and cell viability was further assessed by fluorescence microscopy using acridine orange/ethidium bromide (AO/EB) staining.

#### 2.7.1. Cell Culture

Non-transformed human lung fibroblast cells (MRC-5, ATCC-CCL-171), obtained from the American Type Culture Collection (ATCC, Manassas, VA, USA), were utilized in this study. The cells were cultured in 25 cm^2^ vented flasks (130189, Thermo Scientific, Waltham, MA, USA) using Dulbecco’s Modified Eagle Medium (DMEM, D5671, Sigma-Aldrich, St. Louis, MO, USA), supplemented with 10% heat-inactivated fetal bovine serum (ECS5000L, EuroClone, Pero, Italy), L-glutamine (ECB3000D, EuroClone), non-essential amino acids (M7145, Sigma-Aldrich), and a penicillin–streptomycin mixture (P4333, Sigma-Aldrich). Cells were maintained under standard culture conditions: a humidified atmosphere containing 5% CO_2_ at 37 °C. Upon reaching 70–80% confluence, cells were subcultured or harvested for experiments using a solution containing 0.05% trypsin (6502, Sigma-Aldrich) and 0.053 mM EDTA (E6758, Sigma-Aldrich).

#### 2.7.2. Hydrogel Extract Preparation

Prepared hydrogels were extracted according to ISO standard 10993-12 [33]. Hydrogel samples, including hydrogel base (1, 2, and 3), hydrogels enriched with lower (4 mg/mL; 1EL, 2EL, and 3EL) and higher (8 mg/mL; 1EH, 2EH, and 3EH) concentrations of grape skin extract were first washed with sterile phosphate-buffered saline (PBS, pH 7.4) and subsequently sterilized by UV irradiation for at least 30 min on each side. Following decontamination, hydrogel samples were incubated in DMEM at a biomaterial-to-medium ratio of 0.2 g/mL. Extraction was carried out in chemically inert containers under aseptic conditions at 37 °C for 24 h with stirring. After extraction, the resulting solutions were collected, filtered through a 0.22 µm membrane filter, and diluted with DMEM medium to final concentrations of 50% and 25% (*v*/*v*).

#### 2.7.3. Extract Cytotoxicity Test

Evaluation of extract cytotoxicity was performed in accordance with the modified ISO standard 10993-5 [34]. Cytotoxic potential was assessed using the 3-(4,5-dimethylthiazol-2-yl)-2,5-diphenyltetrazolium bromide (MTT) assay, as described by Mosmann [35]. MRC-5 cells in the exponential growth phase were seeded in 96-well plates (130188, Thermo Scientific, Waltham, MA, USA) at a density of 3 × 10^3^ cells per well. Following overnight adherence, cells were treated with 200 µL of hydrogel extracts at concentrations of 100%, 50%, and 25% for 24 h. Cells incubated in supplemented DMEM without hydrogel extracts served as the untreated (negative) control, whereas cells treated with Tween 20 at concentrations of 0.1%, 0.05%, and 0.025% were used as the positive control.

#### 2.7.4. Direct Cytotoxicity Test

MRC-5 cells were seeded in 6-well plates (130184, Thermo Scientific, Waltham, MA, USA) at a density of 2 × 10^5^ cells per well and incubated overnight to facilitate attachment. Hydrogel samples (0.4 g) were decontaminated as previously described, then carefully placed directly onto the cell monolayer in each corresponding well. The cells were incubated with hydrogels for 24 h under standard culture conditions. The untreated (negative) control consisted of wells containing cells in supplemented DMEM only, while the positive control was represented by wells treated with 0.1% Tween 20. Cytotoxic capacity was determined via an MTT assay.

#### 2.7.5. MTT Assay

After 24 h of treatment under terms of either extract or direct cytotoxic test, the medium was replaced with fresh MTT solution (M5655-5X1G, Sigma-Aldrich) at a final concentration of 0.5 mg/mL, followed by incubation for at least 2 h under standard culture conditions. Subsequently, the MTT solution was removed, and the resulting purple formazan crystals were dissolved in dimethyl sulfoxide (DMSO, D8418-500ML, Sigma-Aldrich) with gentle stirring. Absorbance was measured at 570 nm using a microplate reader (BioTek Epoch Microplate Spectrophotometer, Agilent, Santa Clara, CA, USA; Software Gen5, version 3.17.16). Cell viability percentage was calculated using the following equation: Cell viability % = 100 × OD_T/_OD_c,_ where *OD_T_* represents the mean value of the OD in treated wells, and *OD_C_* represents the mean value of the OD in untreated wells (negative control). Each experiment was conducted in triplicate and repeated three times. Results were presented as an average value with standard deviation (SD).

#### 2.7.6. Confluency and Morphology Analysis

The morphological characterization and confluency assessment of adherent cells were performed using inverted bright-field microscopy combined with quantitative analysis of high-resolution digital micrographs in the ImageJ software environment (NIH, Bethesda, MD, USA; software version 1.54p, https://imagej.net/ij/, accessed on 3 March 2025). Cells were cultured under standard conditions (37 °C, 5% CO_2_, humidified atmosphere) and observed following treatment in Section 2.7.3 and Section 2.7.4. Images were captured by the 2020 S-EYE Setup Microscope camera with S-EYE_Setup-1.6.0.11 software using a trinocular inverted fluorescent microscope (FLUO500T, Gramma Libero, Ljubljana, Slovenia) at a magnification of 20× and saved in TIFF format for subsequent analysis.

#### 2.7.7. Cell Viability Staining

Cell viability was assessed using a dual-fluorescence staining method with acridine orange (AO) and ethidium bromide (EB), as previously described [36]. After treatment in Section 2.7.3, adherent cells were washed with PBS (pH 7.4) to remove non-adherent or dead cells. A staining solution containing AO (10 µg/mL, A1301, Invitrogen, Waltham, MA, USA) and EB (10 µg/mL, J67270.14, Thermo Scientific, Waltham, MA, USA) in PBS was freshly prepared and added directly to the cell monolayer at a final concentration of 1 µg/mL. Following staining, cells were immediately observed using a trinocular inverted fluorescent microscope (FLUO500T, Gramma Libero, Ljubljana, Slovenia) equipped with appropriate filters (excitation ~488 nm, emission ~530 nm for AO; and excitation ~510–550 nm, emission ~600 nm for EB), at a magnification of 20× and captured in TIFF format by the 2020 S-EYE Setup Microscope camera with S-EYE_Setup-1.6.0.11 software. Quantification of fluorescence signal was performed on ImageJ (NIH, Bethesda, MD, USA; software version 1.54k) as previously described [37]. The mean fluorescence intensity (MFI) ratio of EB and AO was calculated as an indicator of cell viability [37]. At least three images per experimental group were analyzed. Results are presented as mean values with standard deviations.

### 2.8. In Vitro Antimicrobial Testing of Hydrogels Loaded with Sustainable Grape Skin Extract

In this study, reference bacterial gram-positive *Staphylococcus aureus* ATCC 25923, gram-negative *Escherichia coli* ATCC 25922, and *Pseudomonas aeruginosa* ATCC 10145, and yeast strain *Candida albicans* ATCC 10231 were used. Microbial strains were enriched in Mueller–Hinton broth (MHB, M1657-500G, Himedia, Maharashtra, India) by overnight incubation at 37 °C, and subsequently maintained on Mueller–Hinton agar (MHA, M1084-500G, Himedia) until use.

#### 2.8.1. Direct Contact Antimicrobial Test

The antimicrobial activity of hydrogel samples was evaluated using a direct contact method. Due to the hydrated and soft nature of hydrogels, the protocol allows direct contact with bacterial suspensions in a liquid environment. The assay was conducted in sterile 24-well plates.

Microbial suspension was made from 18 h-culture and standardized to 0.5 McFarland, followed by serial dilutions to obtain a final concentration of approximately 5 × 10^5^ CFU/mL. Decontaminated hydrogel (Section 2.7.2) samples, including hydrogel base (1, 2, and 3), hydrogels enriched with lower (4 mg/mL; 1EL, 2EL, and 3EL) and higher (8 mg/mL; 1EH, 2EH, and 3EH) concentration of grape skin extract, were placed in wells containing 2 mL of the microbial suspension. Untreated (negative) control wells were microbes in MHB only, while the antibiotic (positive) control gentamicin was used for Gram-negative bacteria, erythromycin for Gram-positive bacteria, and fluconazole for fungi. Plates were incubated at 37 °C for 24 h. Subsequently, microbial growth was assessed spectrophotometrically by measuring optical density at 600 nm (OD_600_) using a microplate reader (BioTek Epoch Microplate Spectrophotometer, Agilent, Santa Clara, CA, USA; Software Gen5, version 3.17.16). The antimicrobial effect of hydrogels was expressed as the percentage of growth inhibition compared to the untreated (negative) control, according to the formula: Inhibition (%) = (1 − OD_sample/OD_control) × 100. Results were reported as mean ± standard deviation.

#### 2.8.2. Live/Dead Staining

Following the 24 h incubation with hydrogel samples (Section 2.8.1), bacterial suspensions were collected from each well and transferred to sterile tubes. The cells were washed three times with 0.9% NaCl by centrifugation (5000 rpm, 10 min) and resuspension to remove residual hydrogel material and broth components. The final bacterial pellet was resuspended in 500 µL of sterile 0.9% NaCl. Viability was assessed using dual-fluorescence staining with AO and EB, which enables differentiation between live and non-viable cells based on membrane integrity, as previously described with modifications [38]. A freshly prepared staining solution was added directly to each suspension to achieve a final concentration of 10 µg/mL for both AO and EB. A 10 µL aliquot of each stained sample was mounted on a microscope slide, covered with a coverslip, and immediately examined at 40× magnification under a trinocular inverted fluorescent microscope (FLUO500T, Gramma Libero, Ljubljana, Slovenia) using appropriate filters. Representative fluorescence images were captured in TIFF format using the 2020 S-EYE Setup Microscope camera and S-EYE_Setup-1.6.0.11 software. Images were processed using the ImageJ software (NIH, Bethesda, MD, USA; software version 1.54k), and the MFI ratio between the EO and AO channels was calculated as a quantitative indicator of bacterial viability. A minimum of three fields per experimental group were analyzed. Data are expressed as mean ± standard deviation.

### 2.9. Antioxidant Properties of Hydrogels Loaded with Sustainable Grape Skin Extract

#### 2.9.1. Sample Preparation

Hydrogel aliquots were prepared using the same procedure described for in vitro extract release study (Section 2.6.5). Following a 24 h release interval—previously validated to achieve equilibrium release kinetics—the supernatant was harvested for subsequent analytical assays.

#### 2.9.2. TPC

The TPC was determined using a microplate adaptation of the Folin–Ciocalteu assay [39]. This method involved preparing a series of gallic acid standards to create a calibration curve, while samples, such as hydrogel supernatants, were diluted to appropriate concentrations if necessary. Each standard and sample was loaded into a 96-well microplate in a volume of 20 μL, followed by the addition of 80 μL of distilled water to ensure uniformity in volume. Subsequently, 20 μL of diluted Folin–Ciocalteu reagent was added to each well, and, after a brief incubation, 80 μL of a 7.5% sodium carbonate solution was introduced to stabilize the color reaction. The mixture was allowed to stand for approximately 30 min to facilitate color development. The absorbance of each well was measured at 765 nm using a microplate reader. By plotting the absorbance values of the standards against their concentrations, a calibration curve was generated, which was used to calculate the TPC of the samples, expressed as micrograms of GAE per gram of xerogel (µg GAE/g xerogel).

#### 2.9.3. DPPH Radical Scavenging Assay

The DPPH radical scavenging activity was assessed using a microplate method adapted for high-throughput analysis. Briefly, test samples and standard (Trolox) were combined with a methanolic DPPH solution (0.004% *w*/*v*) in 96-well plates, with reaction volumes standardized to 200 µL per well. After incubation at room temperature for 30 min in the dark, absorbance was measured at 515 nm. Radical scavenging activity was calculated as a percentage relative to a negative control (DPPH + solvent) to determine antioxidant capacity. The SC_50_ was determined for each sample. This method enabled efficient screening with minimal reagent consumption, validated against established standards to ensure reproducibility [40].

#### 2.9.4. ABTS Radical Cation Decolorization Assay

To determine antioxidant capacity using the ABTS assay, we employed a procedure where ABTS+- radicals were generated by reacting an ABTS stock solution with potassium persulfate in the dark for 16 h according to a previously published protocol. The ABTS+- solution was then diluted to achieve an initial absorbance of approximately 0.700 ± 0.020 at 734 nm. In a microplate setup, 20 µL of each sample or Trolox standard was mixed with 180 µL of the diluted ABTS+- solution, and absorbance was measured at 734 nm within a minute. All measurements were conducted in triplicate to ensure reproducibility [40,41,42].

### 2.10. Statistical Analysis

Statistical analysis was conducted using IBM SPSS Statistics version 20.0 for Windows. The normality of data distribution was assessed using the Shapiro–Wilk test. For data that met the assumption of normality, homogeneity of variances was subsequently evaluated using Levene’s test. Results are presented as mean ± standard deviation (M ± SD). Group differences were analyzed using one-way analysis of variance (ANOVA), followed by post hoc comparisons with the Bonferroni correction. A *p*-value less than 0.05 was considered statistically significant, while *p* < 0.01 was considered highly significant [43].

## 3. Results and Discussion

### 3.1. Extraction Yield, Total Phenolic Content, and Antioxidant Activity of Grape Skin Extract

The selection of extraction techniques is essential for maximizing the potential of bioactive compounds derived from plant materials, such as grape skins. Conventional extraction techniques often use organic solvents and high temperatures. Despite their efficiency, these methods raise serious safety and environmental concerns. Consequently, green alternatives based on the principles of green chemistry have been developed, emphasizing the use of environmentally friendly solvents and processes. Among them, the UAE has become known as a very successful method for isolating bioactive substances. UAE offers a safer and more environmentally friendly alternative to conventional extraction techniques when paired with green solvents such as water, ethanol, and ethyl acetate [16,17,18].

This involved the extraction of bioactive phenolic components from the skins of Cabernet Sauvignon grape type utilizing UAE. The extraction conditions were meticulously optimized to achieve the maximum yield and TPC [16,17,18]. The obtained ethyl acetate extract-E was further subjected to detailed tests of antioxidant capacity and phenolic compounds content.

The quantitative analysis of phenolic compounds conducted through the Folin–Ciocalteu method indicated a significant concentration of total phenolics, measured at 7.9 mg GAE/g E (Table 2), highlighting the extract’s abundance in bioactive phenolic constituents. These compounds are well acknowledged for their diverse biological functions, chiefly influenced by their antioxidant and anti-inflammatory properties [16].

The antioxidant properties were evaluated using various complementary assays to ensure a thorough assessment of their effectiveness (Table 2).

Grape skin extract exhibited significant antioxidant activity, as evidenced by SC50 values of 34.06 µg/mL in the DPPH assay and 17.01 µg/mL in the ABTS assay (Table 2). In comparison, Shiraz extracts have shown even better antioxidative potential with SC50 values of 24.48 µg/mL in the DPPH assay and 5.25 µg/mL in the ABTS assay [16]. The values identified classify the obtained extract as a significant source of polyphenols, proficient in neutralizing diverse forms of free radicals. The observed lower SC_50_ value in the ABTS assay implies greater efficacy in scavenging hydrophilic radicals, which indicates the existence of phenolic compounds that exhibit a higher affinity for aqueous environments.

The antioxidant potential was further validated through the FRAP assay, which yielded an absorbance value of 0.1864 at 700 nm for E, clearly demonstrating a strong ability to reduce ferric ions (Fe^3+^) to ferrous ions (Fe^2+^). The capacity for reduction enhances the antioxidant efficacy of the phenolic components found in the extract, illustrating the possible protective benefits against oxidative harm in biological systems. The elevated phenolic content is closely associated with the antioxidant activity observed, offering a biochemical explanation for the notable bioactivity exhibited in the assays.

### 3.2. HPLC Analysis of Grape Skin Extract

The results of HPLC analysis of ethyl acetate extract of grape skin (E) clearly show that it is a valuable source of natural antioxidants and anti-inflammatory compounds. Analysis of E extract showed that it contains a significant concentration of bioactive phenolic substances. The corresponding chromatograms are given in the supplementary material (Figures S1–S3).

Significant concentrations of gallic acid (566 mg × 10^−2^/g), chlorogenic acid (302 mg × 10^−2^/g), syringic acid (119 mg × 10^−2^/g), epicatechin (5415 mg × 10^−2^/g), and myricetin (908 mg × 10^−2^/g) were found. Previous studies have shown that these compounds exhibit antioxidant, anti-inflammatory, and antimicrobial properties [44,45,46,47], and that they promote wound healing.

More details about the content of individual components of the tested extracts can be found in Table S1.

Considering these findings, it is clear that the Cabernet Sauvignon grape skin extract represents a source of natural compounds that is worthy of attention for both pharmaceutical and nutraceutical use, especially in formulations designed to alleviate oxidative stress and inflammation-related conditions. Given this, this obtained extract presents significant potential for integration into hydrogel formulations that would be suitable for the treatment of chronic wounds.

### 3.3. Physico-Chemical Characterization of Hydrogels Loaded with Sustainable Grape Skin Extract

#### 3.3.1. Swelling Properties

**Figure 1:**
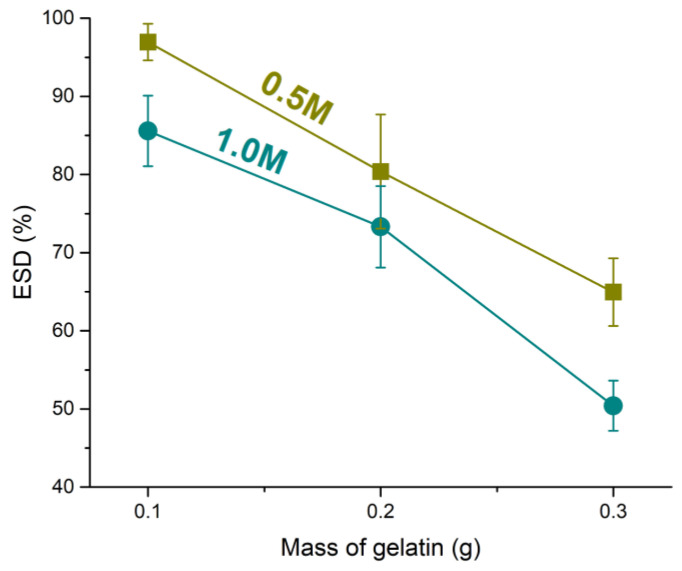
Dependence of the equilibrium swelling degree of hydrogels on gelatin content and CaCl_2_ solution concentration.

One of the key factors influencing the mechanical properties of hydrogels is their propensity to swell. Hydration and water content, often expressed as the ESD, are critical properties of hydrogels, influencing both their biological performance and mechanical behavior. Water within the hydrogel matrix enhances biocompatibility, reduces friction, and facilitates load transfer throughout the structure. Importantly, ESD also serves as an indicator of the degree of crosslinking, as higher crosslinking typically corresponds to lower water uptake. In the current study, the modification of the gelatin–alginate ratio, through changes in the gelatin concentration with constant sodium alginate levels, influenced the water absorption capacity of the hydrogels. As shown in Figure 1, increasing the gelatin content in the hydrogels resulted in a noticeable decrease in ESD, likely due to the formation of a denser and more crosslinked network. This effect can be attributed to the interaction between gelatin and alginate molecules through hydrogen bonding, which enhances structural compactness and limits water absorption. Numerous studies support the fact that sodium alginate, with its negatively charged carboxylate functional groups, creates an electrostatic repulsive force within the network, which enhances the hydrogel’s water uptake properties in concentration up to 4% [48]. Although alginate hydrogels exhibit rapid degradation and a high swelling profile, which can be attributed to their reversible physical crosslinking by Ca^2+^ ions, the addition of gelatin to the alginate system alters swelling properties [49]. Similarly, in our research, increasing the concentration of CaCl_2_ further reduced the swelling capacity of the hydrogels, as the higher availability of calcium ions promoted more extensive ionic crosslinking with alginate chains. These findings highlight the dual role of gelatin and CaCl_2_ concentration in modulating the network structure and hydration properties of alginate-gelatin hydrogels. These data provide valuable insight into the capacity of hydrogels to absorb water or other fluids, which is of crucial importance for their clinical applications, such as in wound healing and tissue engineering [50].

#### 3.3.2. Microstructure

**Figure 2:**
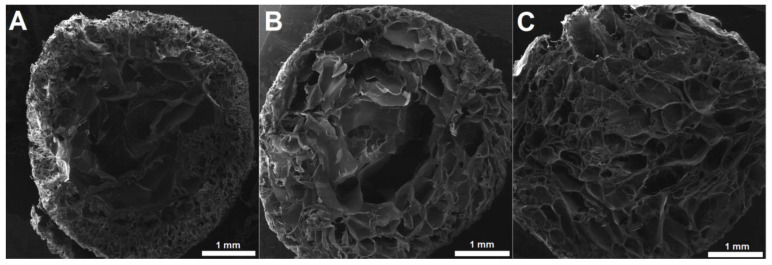
Cross-section micrographs of freeze-dried hydrogels: Alg/Gel-1 (**A**), Alg/Gel-2 (**B**), and Alg/Gel-3 (**C**).

SEM analysis of the cross-sections of Ca^2+^-crosslinked alginate/gelatin hydrogels revealed a distinct core–shell morphology in formulations with lower gelatin content. The outer region of the hydrogels exhibited a compact structure with small, uniform pores measuring up to 100 µm (Figure 2A). In contrast, the interior displayed significantly larger, interconnected pores ranging from 300 to 800 µm (Figure 2A,B). This core–shell structure became progressively less pronounced with increasing gelatin concentration. At the highest gelatin content (3%), the outer shell was nearly absent, and the pore distribution became more homogeneous throughout the entire cross-section, with average pore sizes of approximately 300 µm (Figure 2C). The formation of a denser outer shell in low-gelatin hydrogels may be attributed to rapid ionotropic gelation of alginate in the outermost layers upon contact with the Ca^2+^ solution, which restricts polymer chain mobility and leads to smaller pores. In lower gelatin formulations, gelatin likely plays a limited role in modulating this effect, allowing a dense surface layer to form. As the gelatin concentration increases, its influence on the network architecture becomes more pronounced. Gelatin can interfere with the alginate crosslinking dynamics by increasing the viscosity of the pre-gel solution and competing for space in the network, resulting in more uniform porosity throughout the sample. Additionally, higher gelatin content may reduce the extent of immediate surface gelation by hindering Ca^2+^ diffusion, leading to a more homogeneous structure with intermediate-sized pores.

#### 3.3.3. Mechanical Properties

**Figure 3:**
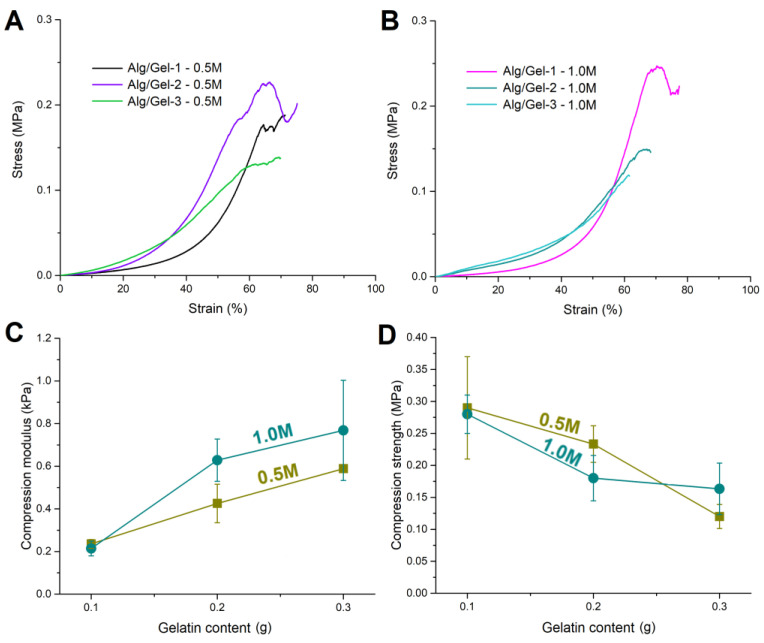
Stress–strain curves of alginate–gelatin hydrogels crosslinked in 0.5 M (**A**) and 1.0 M CaCl_2_ solutions (**B**). The influence of gelatin content under different crosslinking conditions on compression modulus (**C**) and compression strength (**D**) of alginate–gelatin hydrogels.

Figure 3A,B present the stress–strain curves of alginate–gelatin hydrogels crosslinked in CaCl_2_ solutions at concentrations of 0.5 M and 1.0 M, respectively. The sodium alginate content was kept constant, while the gelatin content varied (0.1 g, 0.2 g, and 0.3 g), corresponding to samples Alg/Gel-1, 2, and 3. The stress–strain curves indicate that increasing the gelatin content reduced the maximum strain at failure, while simultaneously increasing the modulus of the hydrogels. As the gelatin content in alginate–gelatin hydrogels increases, the hydrogels become stiffer but can stretch less before breaking. This change in mechanical properties is significant because it allows for the customization of the hydrogels’ behavior depending on the intended application [51]. The importance of performing this analysis is reflected in the fact that high material stiffness can lead to significant tissue deformation, which might impair the regeneration process and cause damage that interrupts tissue continuity [52].

Figure 3C illustrates the relationship between the compression modulus and gelatin content under both crosslinking conditions. A positive correlation is observed, with the compression modulus rising alongside gelatin content. Hydrogels crosslinked in 1.0 M CaCl_2_ consistently exhibited higher compression moduli than those immersed in the 0.5 M solution, indicating that the stronger ionic crosslinking induced by the higher CaCl_2_ concentration resulted in stiffer network structures. Notably, the trend in compression modulus appeared inversely related to the ESD, reinforcing the concept that denser, more tightly crosslinked networks exhibit lower swelling capacities and greater mechanical stiffness. Additionally, Figure 3D illustrates the relationship between compressive strength and gelatin content. Unlike the compressive modulus, the compressive strength generally decreased with increasing gelatin content under both crosslinking conditions. This decline could be attributed to the interference of gelatin with alginate crosslinking, which reduced overall network cohesion. Although the swelling behavior and compressive modulus suggest a higher crosslinking density in hydrogels with gelatin, the distribution of crosslinks was likely non-uniform. An increase in gelatin content may have hindered the diffusion of Ca^2+^ ions, leading to preferential crosslinking in the outer layers of the hydrogels, as evidenced by SEM micrographs (Figure 2). This phenomenon, which is commonly observed in alginate hydrogels, was likely more pronounced in this system [40,44]. Consequently, the hydrogels exhibited a lower ESD and a higher modulus. However, due to the uneven crosslink distribution, primarily concentrated in the outer layers, the hydrogels developed a stiff outer shell while remaining mechanically weaker in the core. In the case of Alg/Gel-1, which exhibited the thickest outer shell, the hydrogel demonstrated the highest compressive strength due to a more uniform distribution of crosslinks throughout its volume. As the gelatin content increased, crosslinking became increasingly concentrated near the surface, contributing to a stiffer shell but a less crosslinked core, ultimately leading to a reduction in overall compressive strength. A similar trend was observed with the crosslinking solution concentration, where a higher Ca^2+^ ion concentration resulted in reduced compressive strength of the hydrogels. These findings underscore the dual role of gelatin in the hydrogel network, where it enhances stiffness while simultaneously compromising the overall load-bearing capacity under compressive stress [52,53].

#### 3.3.4. FTIR Analysis

**Figure 4:**
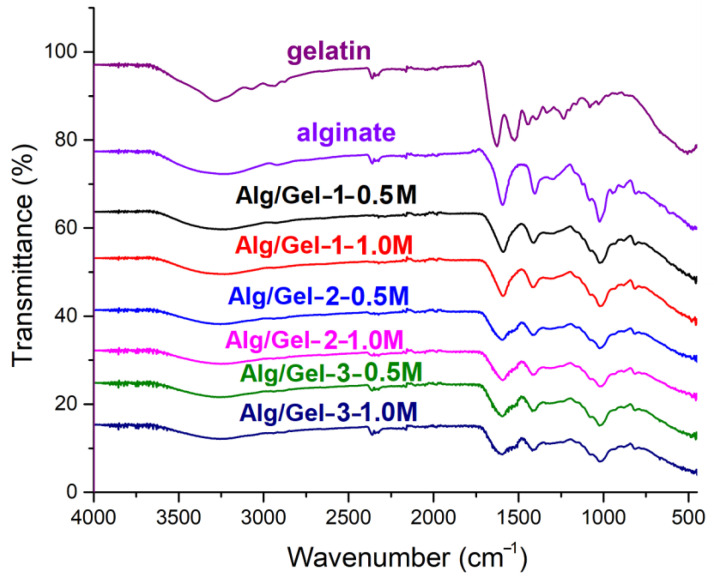
FTIR spectra of alginate–gelatin hydrogels with various % of gelatin and CaCl_2_.

The FTIR spectra of pristine gelatin and sodium alginate are presented in Figure 4. The gelatin spectrum featured characteristic peaks at 1628, 1522, and 1235 cm^−1^ corresponding to amide I (C=O stretching), amide II (N–H bending and C–N stretching), and amide III vibrations, respectively, associated with the polypeptide backbone. A broad band at 3275 cm^−1^ was assigned to overlapping N–H and O–H stretching vibrations, while a peak at 3071 cm^−1^ was attributed to free N–H stretching. Peaks at 2935 and 2874 cm^−1^ were assigned to C–H stretching of aliphatic chains. In contrast, the FTIR spectrum of the pure sodium alginate hydrogel exhibited a broad O–H stretching band at 3240 cm^−1^ and a C–H stretching band at 2921 cm^−1^. Prominent peaks at 1593 cm^−1^ and 1403 cm^−1^ were attributed to the asymmetric and symmetric stretching vibrations of the carboxylate groups (COO^−^), while bands at 1023 cm^−1^ and 1081 cm^−1^ corresponded to C–O stretching. Additional peaks at 946 cm^−1^ and 883 cm^−1^ confirmed the presence of guluronic and mannuronic acid units, and a peak at 810 cm^−1^ reflected Na–O stretching in sodium alginate. Upon blending with gelatin and ionic crosslinking using Ca^2+^ ions, notable spectral changes occurred that indicate enhanced intermolecular interactions and network formation. In the Alg/Gel-1 sample, the O–H/N–H stretching band appeared at 3239 cm^−1^, and the carboxylate peaks at 1590 and 1411 cm^−1^ remained sharp, suggesting limited interaction at lower gelatin content. However, as the gelatin concentration increased, the O–H/N–H region shifted to 3252 cm^−1^ and became broader, while the carboxylate bands at 1594 and 1411–1413 cm^−1^ became less defined. These changes indicate increasing hydrogen bonding between hydroxyl and amine groups, as well as electrostatic interactions between alginate’s COO^−^ groups and gelatin’s NH_3_^+^ groups. Moreover, the ionic crosslinking with Ca^2+^ likely intensified these effects by promoting COO^−^–Ca^2+^–COO^−^ bridging, further altering the vibrational environment of carboxylate groups and contributing to the observed shifts and broadening. The persistence of the C–O stretching bands at 1023–1024 cm^−1^ suggests that the alginate backbone structure remains chemically stable. Collectively, these findings confirm that the gelatin–alginate hydrogels form via non-covalent mechanisms—primarily hydrogen bonding, electrostatic interactions, and calcium-mediated ionic crosslinking.

#### 3.3.5. In Vitro Extract Release from Hydrogels

**Figure 5:**
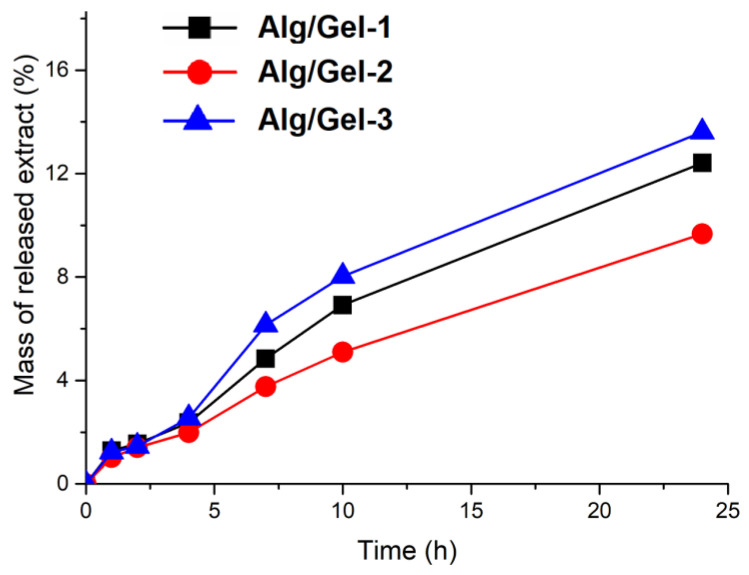
Mass of released extracts (%) as a function of time for E extract.

After completing tests to optimize hydrogel properties, including stiffness, swelling behavior, and the effects of gelatin content and crosslinking conditions, we selected formulations crosslinked with a 1.0 M CaCl_2_ solution for further incorporation of grape skin extract. In vitro release study demonstrated that the grape skin extract was released rapidly during the first 10 h, followed by a slower, sustained release over the subsequent 14 h (Figure 5). This pattern, which involves an initial rapid release under physiological conditions (temperature 37 °C; pH 7.4) followed by a sustained one, is beneficial for biomedical uses like drug delivery, where maintaining a controlled release is key to providing both fast-acting and lasting therapeutic effects [54,55]. Our developed hydrogel, which releases the grape skin extract over 24 h, demonstrated suitable properties that encouraged further exploration of antioxidant and antimicrobial activity. The variation in extract release among the hydrogel formulations can be attributed to the interplay between crosslinking density, structural heterogeneity, and the gradual degradation of gelatin at 37 °C in PBS. Although Alg/Gel-3 exhibited the lowest swelling capacity and was initially expected to release the extract more slowly, it showed the highest release rate. This behavior was primarily due to its high gelatin content, which led to an accelerated degradation under physiological conditions, enhancing internal porosity over time. Alg/Gel-1 demonstrated the second-highest release rate; its high swelling ability and loosely crosslinked network facilitated diffusion, but its low gelatin content limited the extent of degradation-driven porosity. In contrast, Alg/Gel-2 showed the slowest release, likely due to a more balanced combination of crosslinking density and gelatin content, which resulted in a denser, more diffusion-restrictive structure with moderate degradability. These results highlight the importance of optimizing the mechanical and structural properties of hydrogels to achieve the desired release kinetics for specific therapeutic applications [5].

#### 3.3.6. Analysis of the Kinetics of Drug Release

The release kinetics of the extract from the Alg/Gel hydrogels were analyzed using the Korsmeyer–Peppas model, which is widely applied to polymeric systems where multiple mechanisms may govern release behavior. The calculated diffusional exponents (n) were 0.79 for Alg/Gel-1, 0.78 for Alg/Gel-2, and 0.76 for Alg/Gel-3 (Table 3). According to the model’s criteria for disc-shaped hydrogels—where n = 0.43 indicates Fickian diffusion and 0.43 < n < 0.85 signifies anomalous (non-Fickian) transport—all three formulations exhibited anomalous diffusion behavior. This indicates a release mechanism governed by both Fickian diffusion and polymer relaxation or degradation processes. Among the formulations, Alg/Gel-1 and Alg/Gel-2 showed similar n values, suggesting comparable contributions from both diffusion and matrix relaxation. Alg/Gel-3, while also within the anomalous range, had a slightly lower n value than the others, which may reflect a stronger initial diffusion component followed by structural breakdown due to its high gelatin content. This interpretation is supported by the relatively rapid increase in cumulative release observed over time for Alg/Gel-3. The coefficients of determination (R^2^) for the model fits were 0.99, 0.99, and 0.98 for Alg/Gel-1, Alg/Gel-2, and Alg/Gel-3, respectively, indicating excellent agreement with the Korsmeyer–Peppas model across all samples. These results reinforce the hypothesis that the release behavior is governed not only by diffusion but also by evolving internal structural changes, particularly those arising from gelatin degradation in formulations with higher gelatin content.

According to the literature data [4], many commercial hydrogels for biomedical applications are primarily composed of synthetic polymers, such as acrylics, polyethylene glycol, polyvinylpyrrolidone, and acrylamide derivatives, often lacking bioactive substances. These synthetic polymer-based hydrogels generally exhibit release profiles governed mainly by passive diffusion through their network structures. In contrast, our hydrogel utilizes natural, biodegradable polymers combined with antioxidant-rich grape skin extract, enabling a controlled release driven by both diffusion and polymer relaxation mechanisms.

### 3.4. Biocompatibility of Hydrogels Loaded with Sustainable Grape Skin Extract

Ensuring biocompatibility is a critical step in the development of any wound dressing. Given the intended biomedical application of the alginate–gelatin hydrogels enriched with grape skin extract, their cytocompatibility was evaluated using standard in vitro assays. Human lung fibroblasts (MRC-5) were selected as a representative normal cell line due to their relevance in tissue repair processes. Both direct contact and extract-based exposure tests were conducted, alongside morphological and membrane integrity assessments. These complementary approaches aimed to confirm that the hydrogels, as well as their released components, do not induce cytotoxic effects or compromise cell structure and function.

**Figure 6:**
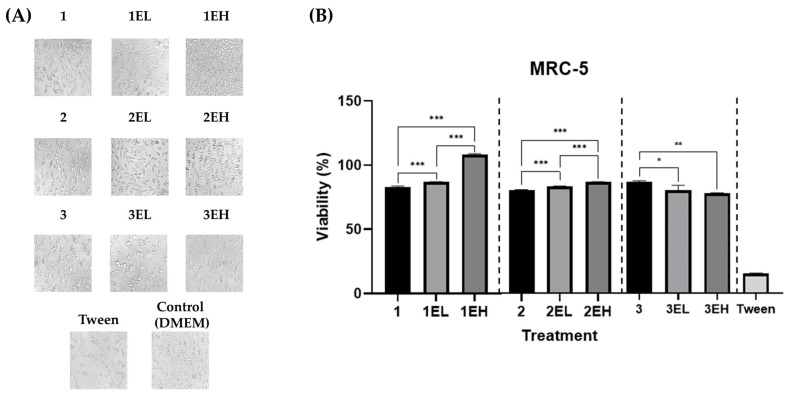
Direct contact cytocompatibility assessment of grape extract-enriched hydrogels on MRC-5 cells: (**A**) Bright-field images, 20× objective, of MRC-5 cells after 24 h exposure to hydrogels base (1, 2, 3), grape skin extract-enriched formulations with lower and higher concentrations (EL and EH respectively), positive control Tween 20, and untreated (negative) control (DMEM). (**B**) MTT assay results showing MRC-5 viability (% of untreated control) after direct contact with hydrogels. Data are presented as M ± SD. Normality and homogeneity of variances were confirmed using the Shapiro–Wilk and Levene’s tests, respectively, prior to ANOVA. Significance: *p* < 0.05 (*), *p* < 0.01 (**), *p* < 0.001 (***).

The biocompatibility of grape skin extract-enriched alginate–gelatin hydrogels was first assessed through direct contact with MRC-5 human lung fibroblasts (Figure 6). After 24 h incubation, cell viability, determined by the MTT assay, remained above 80% across all formulations, indicating the absence of cytotoxicity. Bright-field microscopy confirmed these findings, revealing normal fibroblast morphology and preserved monolayer integrity across all treated groups. Cells remained well-spread, elongated, and confluent, similar to the untreated control. No signs of membrane blebbing, rounding, or detachment were observed, further supporting the non-toxic nature of the materials under direct exposure conditions.

**Figure 7:**
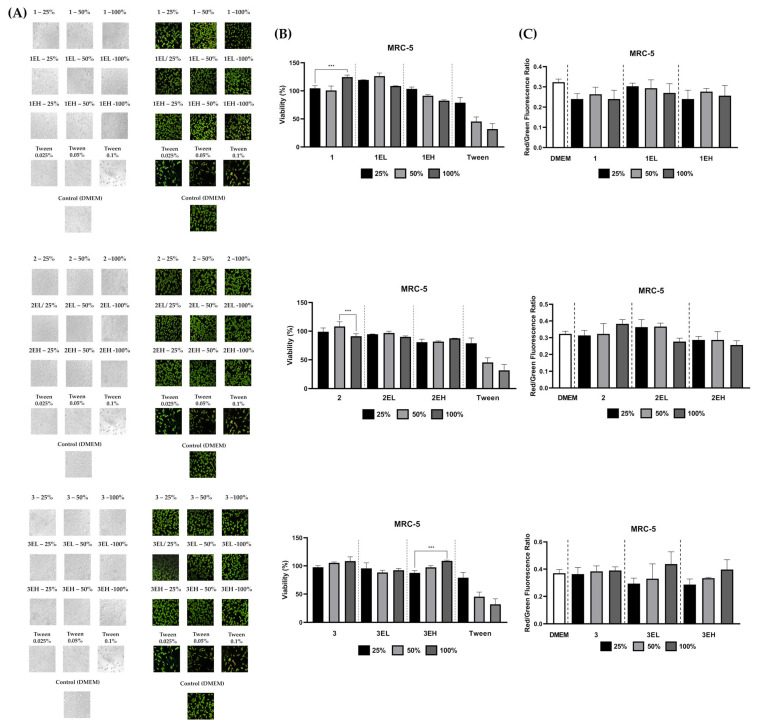
Cytocompatibility of grape-based hydrogel extracts on MRC-5 cells: (**A**) Bright-field and live/dead fluorescence microscopy images, 20× objective, of MRC-5 fibroblasts after 24 h exposure to extracts released from hydrogels base (1, 2, 3), grape skin extract-enriched formulations with lower and higher concentration (EL and EH respectively) at 25%, 50%, and 100% concentrations. Teen 20 was used as a positive control at 0.025%, 0.05%, and 0.1% concentrations. DMEM was used as the untreated (negative) control. (**B**) MTT assay showing cell viability expressed as a percentage relative to the untreated (negative) control. (**C**) Red/green fluorescence ratio after EO/AB staining as an indicator of cell membrane integrity. Data are presented as M ± SD. Normality and homogeneity of variances were confirmed using the Shapiro–Wilk and Levene’s tests, respectively, prior to ANOVA. Statistical significance is indicated as *p* < 0.001 (***).

To evaluate the potential cytotoxicity of components leached from hydrogels, MRC-5 fibroblasts were treated with hydrogel extracts at 25%, 50%, and 100% concentrations (Figure 7). Cell viability remained high across all tested concentrations, particularly at 25% and 50%, with no formulation causing a significant drop below 80%. A mild, concentration-dependent decline in viability was observed at 100% for a few high-extract samples (e.g., 3EH), but values remained within acceptable biocompatibility thresholds per ISO 10993-5 standards [34].

Microscopic analysis post-extract treatment revealed a dose-dependent effect on cell density and confluency but no dramatic changes in cell morphology. At higher extract concentrations, cells appeared slightly less dense, yet retained normal morphology—elongated, spindle-shaped, and firmly attached—indicating limited adverse effects.

Live/dead staining using acridine orange and ethidium bromide (AO/EB) further confirmed these findings (Figure 7). Fluorescence microscopy showed predominantly green-fluorescing cells, indicating intact membranes and high viability. The red/green (EB/AO) fluorescence ratio remained low across all treatment groups, with only minor increases at 100% extract concentration, suggesting minimal membrane compromise. These results corroborate the MTT assay, supporting the conclusion that the extracts are non-cytotoxic and do not induce membrane damage under the tested conditions.

Collectively, both direct and extract-based assays confirmed that the developed hydrogels are highly biocompatible. These findings align with previous studies reporting favorable cytocompatibility of polyphenol-enriched formulations [56,57]. Plant-derived polyphenols, when properly formulated, have demonstrated low cytotoxicity and even regenerative potential in wound healing models [58]. Moreover, our results reaffirm the established biocompatibility of alginate–gelatin hydrogel matrices as biomedical scaffolds. Alginate and gelatin are well-known for their biological safety and have been widely used in tissue engineering due to their inherent biocompatibility and ability to support cell adhesion and growth [59].

### 3.5. Antimicrobial Activity of Hydrogels Loaded with Sustainable Grape Skin Extract

**Figure 8:**
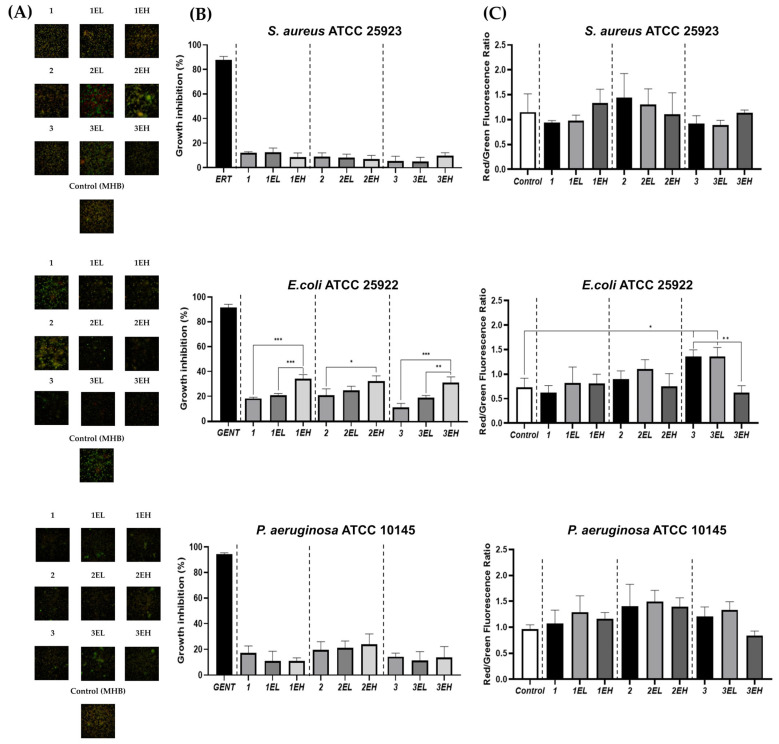
Antimicrobial effects of grape-based hydrogels against *S. aureus, E. coli*, and *P. aeruginosa* in direct contact assay: (**A**) Fluorescence images, 40× objective, of *S. aureus ATCC 25923*, *E. coli ATCC 25922*, and *P. aeruginosa ATCC 10145* after 24 h direct contact with hydrogel base (1, 2, 3), extract-enriched formulations with lower and higher concentration of grape skin extract (EL and EH, respectively), stained with AO/EB. Live bacteria appear green; dead/damaged bacteria appear red. MHB was used as an untreated (negative) control. (**B**) Bacterial growth inhibition (% of untreated control) after direct hydrogel contact was determined by measuring OD_600_. ERT = erythromycin (*S. aureus* control), GENT = gentamicin (*E. coli, P. aeruginosa* controls). (**C**) Red/green fluorescence ratio after AO/EB staining indicating bacterial membrane integrity loss. Data are presented as M ± SD. Normality and homogeneity of variances were confirmed using the Shapiro–Wilk and Levene’s tests, respectively, prior to ANOVA. Significance: *p* < 0.05 (*), *p* < 0.01 (**), *p* < 0.001 (***).

The antimicrobial activity of alginate–gelatin hydrogels enriched with grape skin extract was assessed against four reference strains: *S. aureus* ATCC 25923, *E. coli* ATCC 25922, *P. aeruginosa* ATCC 10145 (Figure 8), and *C. albicans* ATCC 10231 (Supp. Figure S5) using direct-contact and AO/EB staining assays.

*E. coli* ATCC 25922 was the most susceptible microorganism. A statistically significant, dose-dependent inhibitory effect was observed only against *E. coli* ATCC 25922 after 24 h of treatment. All high-extract hydrogels (EHs) significantly inhibited their growth by approximately 35%, whereas base and low-extract formulations (ELs) had minimal effect. Interestingly, AO/EB staining of *E. coli* ATCC 25922 showed low EB/AO fluorescence ratios, indicating preserved membrane integrity despite moderate growth inhibition. This suggests that the antimicrobial mechanism against *E. coli* ATCC 25922 is likely bacteriostatic, involving metabolic interference rather than direct lysis.

For *S. aureus* ATCC 25923 and *P. aeruginosa* ATCC 10145, growth inhibition was limited and not statistically significant. Slight increases in EB/AO ratios were noted in both bacteria, indicating some degree of membrane compromise. These bacteria are generally more resistant due to thick peptidoglycan walls (*S. aureus*) and multiple efflux and defense systems (*P. aeruginosa*), which limit polyphenol efficacy [60].

Surprisingly, *C. albicans* ATCC 10231 exhibited increased growth in the presence of extract-containing hydrogels, suggesting a stimulatory effect of grape extract components, and this strain was, therefore, excluded from further antimicrobial evaluation. Although ethyl acetate extraction minimizes hydrophilic nutrients that commonly support fungal proliferation, certain bioactive compounds, such as polyphenols, amino acids, or sugars, present in the extract might have supported fungal growth under the tested conditions [61,62,63]. The current hydrogel formulation is designed for use on clean or bacterially contaminated wounds, rather than wounds susceptible to fungal colonization. It is important to highlight that the risk of fungal colonization on the skin in healthy, immunocompetent individuals is minimal [64]. Given that hydrogel exhibited stimulatory effects on *C. albicans* growth, it is contraindicated for use in immunocompromised patients, individuals at high risk of fungal infections, and those with active fungal infections. To address this concern, the incorporation of natural antifungal agents with proven activity against *Candida* species into the hydrogel matrix will be considered in future formulation development.

The antimicrobial properties of the hydrogels can be attributed to phenolic constituents identified in the tested grape skin extract, such as gallic acid, epicatechin, and myricetin, known to affect bacterial metabolism, inhibit enzymes, and induce oxidative stress [60]. The selectivity toward *E. coli* aligns with previous studies on polyphenol-rich plant extracts and reflects the vulnerability of Gram-negative membranes to phenolic interference [65]. Importantly, many commercial hydrogels, typically formulated with synthetic polymers, do not consistently demonstrate antimicrobial efficacy [4]. This is often because antimicrobial activity is not a primary design focus, and these products either lack natural bioactive compounds or have not undergone comprehensive antimicrobial testing [4]. Previous research underscores the strong efficacy of gelatin–alginate-based systems; however, direct comparison with our hydrogel incorporating grape skin extract is limited, as those studies rely on antibiotics while we utilize a natural antimicrobial alternative. Earlier studies have investigated gelatin–alginate hydrogels and sponges for wound care applications, demonstrating notable antimicrobial properties. For example, chemically cross-linked gelatin–alginate hydrogels without bioactive natural extracts exhibited broad-spectrum antimicrobial activity against pathogens such as *E. coli*, *S. aureus*, and *P. aeruginosa* [5]. Additionally, gelatin–alginate sponges loaded with the antibiotic tetracycline hydrochloride showed significant antibacterial effects against *E. coli* and *S. aureus* [12]. Furthermore, a 3D bioprinted hydrogel dressing composed of the same polymers and loaded with ciprofloxacin hydrochloride was developed to provide rapid and effective antimicrobial action for wound care [66]. Our natural, sustainable formulation demonstrates selective antimicrobial activity, especially against *E. coli*, combined with excellent biocompatibility, positioning it as a promising alternative for infection-prone wound application.

### 3.6. Antioxidant Activity of Hydrogels Loaded with Sustainable Grape Skin Extract

Assessing the antioxidant properties of sustainable hydrogels enriched with grape skin extract is an important aspect for understanding the therapeutic potential, as oxidative stress plays a key role in delaying wound healing by damaging cells and extracellular matrix components [67]. A comparative analysis of the antioxidant activity of different combinations of hydrogels with grape skin extract and standard (Trolox) is presented in Table 4. Incorporating antioxidants into hydrogel systems may help neutralize reactive oxygen species at the wound site, thereby promoting tissue regeneration and reducing inflammation [68].

The antioxidant activity of the developed hydrogels was primarily attributed to the incorporated grape skin extract rich in bioactive compounds, consistent with its previously described and discussed antioxidant potential (Section 3.1). Base formulations without the extract (samples 1, 2, and 3) exhibited negligible activity, as reflected in high SC_50_ values for DPPH (332.16–348.91 µg/mL) and ABTS (296.89–298.70 µg/mL), confirming the extract as the principal contributor. A clear dose-dependent relationship was observed, where hydrogels with low (EL) and high (EH) extract content demonstrated significantly enhanced antioxidant activity, with SC50 values decreasing notably in 1EH (200.71 ± 21.93 µg/mL for DPPH; 169.86 ± 19.91 µg/mL for ABTS) and 3EH (215.18 ± 21.78 µg/mL for DPPH; 153.99 ± 19.09 µg/mL for ABTS). This trend was consistent across both DPPH and ABTS assays and paralleled increasing TPC, reaching over 230 mg GAE/g E in EH formulations. Additionally, the antioxidant performance was modulated by hydrogel properties, particularly the release profile, with formulations exhibiting sustained release maintaining prolonged antioxidant effects. While gelatin and alginate lacked inherent antioxidant activity, their structural and biocompatible roles can contribute to wound healing by preserving moisture, enabling gas exchange, and supporting cell migration and adhesion [5].

Our developed hydrogel offers distinct advantages through the unique combination of biopolymers and grape skin extract, a natural source of bioactive compounds. In addition, the entire formulation represents a sustainable approach by utilizing renewable materials and environmentally friendly processes, aligning with current trends in green and eco-conscious biomedical product development. Unlike many commercial hydrogels that rely on synthetic polymers and antibiotics that can contribute to antibiotic resistance [4], our formulation presents a promising natural alternative with a better safety profile. However, limitations include a potentially narrower antimicrobial spectrum compared to conventional antibiotic-loaded materials and the need for further in vivo studies to confirm long-term safety and efficacy. Additionally, this hydrogel is not intended for use in immunocompromised patients, as careful consideration is necessary to mitigate the risk of opportunistic infections.

## 4. Conclusions

In conclusion, this study presents the first comprehensive evaluation of alginate–gelatin hydrogels loaded with grape skin extract, offering a novel and sustainable strategy for wound care. Increasing gelatin content significantly enhanced stiffness and crosslink density, while reducing swelling and promoting extract release through degradation-induced porosity. Gelatin acted as a key structural and functional modifier, enabling precise tuning of hydrogel performance. The inclusion of grape skin extract conferred dose-dependent antioxidant activity, high biocompatibility, and selective antimicrobial effects, particularly against *E. coli*, attributed to major phenolics such as caffeic acid, naringin, and epicatechin. Altogether, these multifunctional, eco-friendly hydrogels demonstrate strong potential as tunable platforms for controlled delivery and regenerative wound healing applications.

## Figures and Tables

**Table 1:** Amounts of substances used for the preparation of different hydrogel combinations.

Formulations	Alg/Gel-1	Alg/Gel-2	Alg/Gel-3
Alginate	0.3 g	0.3 g	0.3 g
Gelatin	0.1 g	0.2 g	0.3 g

**Table 2:** Antioxidant activity and TPC of investigated grape skin extracts and standards.

Investigated Extract and Standards	Yield (%)	DPPH SC_50_ (µg/mL)	ABTS SC_50_ (µg/mL)	FRAP (A_700nm_)	TPC (mg GAE/g E)
E	7.8	34.0 ± 0.6	17.0 ± 0.6	0.1864 ± 0.0049	7.9 ± 0.2
NDGA	/	0.5 ± 0.1	ND	ND	/
Quercetin	/	1.9 ± 0.1	ND	ND	/
Ascorbic acid	/	18.0 ± 0.1	31.1 ± 0.1	0.1249 ± 0.0022	/

E-Grape skin extract obtained from Cabernet Sauvignon; abbreviation—NDGA—nordihydroguaiaretic acid; Not Determined—ND.

**Table 3:** Kinetic parameters and coefficients of determination for drug release profiles fitted to the Korsmeyer–Peppas model.

Sample	k	n	R^2^
Alg/Gel-1	1.02	0.79	0.99
Alg/Gel-2	0.81	0.78	0.99
Alg/Gel-3	1.24	0.76	0.98

**Table 4:** Antioxidant activity and TPC of investigated hydrogels loaded with grape skin extracts and standards.

InvestigatedHydrogels and Standards	DPPHSC_50_ (µg/mL)	ABTSSC_50_ (µg/mL)	TPC(µg GAE/g E)
1	332.16 ± 33.49 ^a^	298.70 ± 29.93 ^a^	4.41 ± 0.01
1EL	279.16 ± 29.09 ^a^	279.05 ± 26.33 ^a^	108.45 ± 13.35 *
1EH	200.71 ± 21.93 ^a^*^#^	169.86 ± 19.91^a^*^#^	233.01 ± 23.24 *^#^
2	341.73 ± 32.55 ^a^	297.99 ± 28.04 ^a^	4.46 ± 0.01
2EL	281.46 ± 29.46 ^a^	249.47 ± 23.77 ^a^	115.10 ± 10.12 *
2EH	201.13 ± 21.25 ^a^*^#^	168.47 ± 18.80 ^a^*^#^	234.29 ± 20.16 *^#^
3	348.91 ± 33.73 ^a^	296.89 ± 28.36 ^a^	4.24 ± 0.019
3EL	288.65 ± 29.77 ^a^	223.67 ± 26.72 ^a^	117.02 ± 12.93 *
3EH	215.18 ± 21.78 ^a^*^#^	153.99 ± 19.09 ^a^*^#^	243.12 ± 21.90 *^#^
Trolox	5.03 ± 0.32	6.90 ± 0.69	/

Hydrogels without extract (1, 2, 3); extract-enriched formulations with lower and higher concentrations (EL and EH, respectively), TPC—total phenolic content; GAE—gallic acid equivalent; data are presented as M ± SD. Normality and homogeneity of variances were confirmed using the Shapiro–Wilk and Levene’s tests, respectively, prior to ANOVA. ^a^—significant difference at the level *p* < 0.01 in comparison with trolox; *—significant difference at the level *p* < 0.05 in comparison with groups 1, 2, and 3; ^#^—significant difference at the level *p* < 0.05 in comparison with lower dose extract (L) groups.

## Data Availability

Data are contained within the article.

## Supplementary Materials

The following supporting information can be downloaded at: https://www.mdpi.com/article/10.3390/pharmaceutics17060790/s1. Figure S1: Chromatogram of E at 254 nm; Figure S2: Chromatogram of E at 280 nm; Figure S3: Chromatogram of E at 360 nm; Figure S4. Positive control in cytocompatibility of Grape-Based Hydrogels Extracts on MRC-5 Cells: (A) Bright-field and live/dead fluorescence microscopy images of MRC-5 fibroblasts after 24 h exposure to different concentrations of Tween 20, DMEM was used as the untreated control. (B) MTT assay showing cell viability expressed as a percentage relative to the control. (C) Red/green fluorescence ratio after EO/AB staining as an indicator of cell membrane integrity. Data are presented as M ± SD. Normality and homogeneity of variances were confirmed using the Shapiro–Wilk and Levene’s tests, respectively, prior to ANOVA. Significance: *p* < 0.05 (*), *p* < 0.01 (**), *p* < 0.001 (***); Figure S5. Antimicrobial Effects of Grape-Based Hydrogels Against C. albicans ATCC 10231 in Direct Contact Assay: Bacterial growth inhibition (%) after direct hydrogel contact determined by measuring OD600. FLC = fluconazole (control). Hydrogels base (1, 2, 3), extract-enriched formulations with lower and higher concentration of grape skin extract (EL and EH respectively). Data are presented as M ± SD. Normality and homogeneity of variances were confirmed using the Shapiro–Wilk and Levene’s tests, respectively, prior to ANOVA. Significance: *p* < 0.05 (*), *p* < 0.01 (**), *p* < 0.001 (***).; Table S1. Phenolic acid and flavonoid content (mg × 10^−2^/g ± SD) in grape sample. Legend: * SD—standard deviation, N/A—not available, DHB—Dihydroxy benzoic acid; E—grape skin extract.
