# Supplementary material for: Development and Optimization of Grape Skin Extract-Loaded Gelatin–Alginate Hydrogels: Assessment of Antioxidant and Antimicrobial Properties
Source: Pharmaceutics. 2025 Jun 17;17(6):790. doi: 10.3390/pharmaceutics17060790 (PMC12196999; doi:10.3390/pharmaceutics17060790)
Supplement: Supplementary file 1 [file pharmaceutics-17-00790-s001.zip › pharmaceutics-3653379-supplementary.pdf]

Supplementary Material

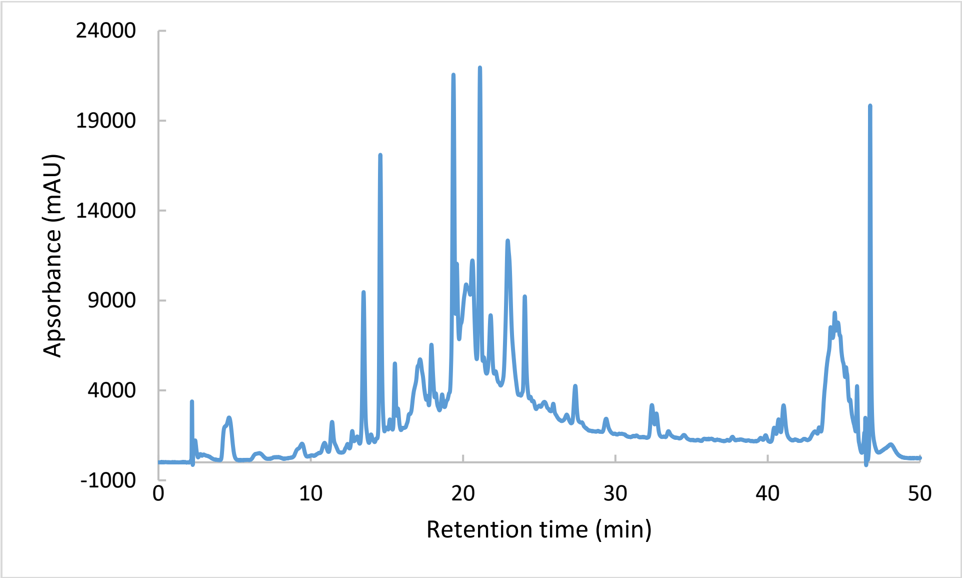

Figure S1: Chromatogram of E at 254 nm

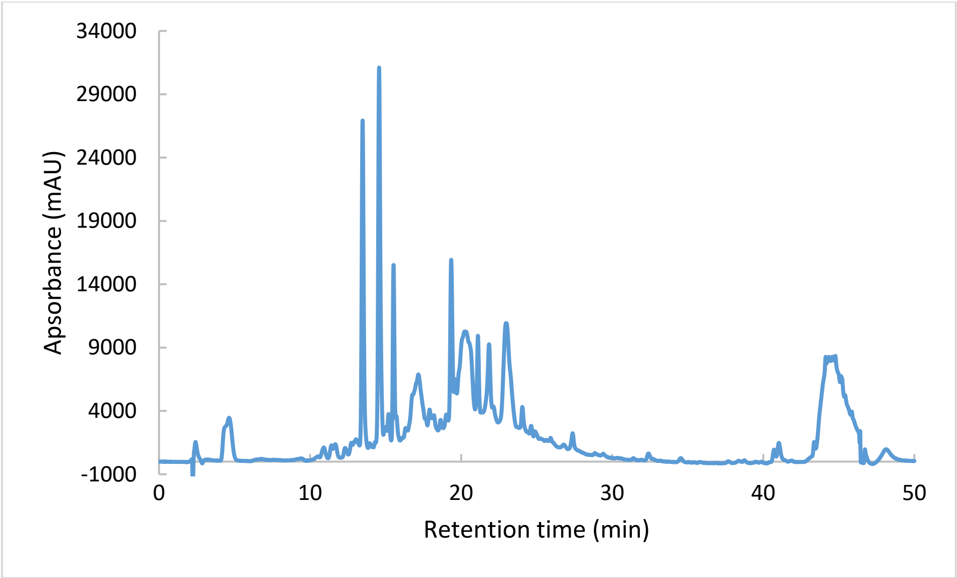

Figure S2: Chromatogram of E at 280 nm

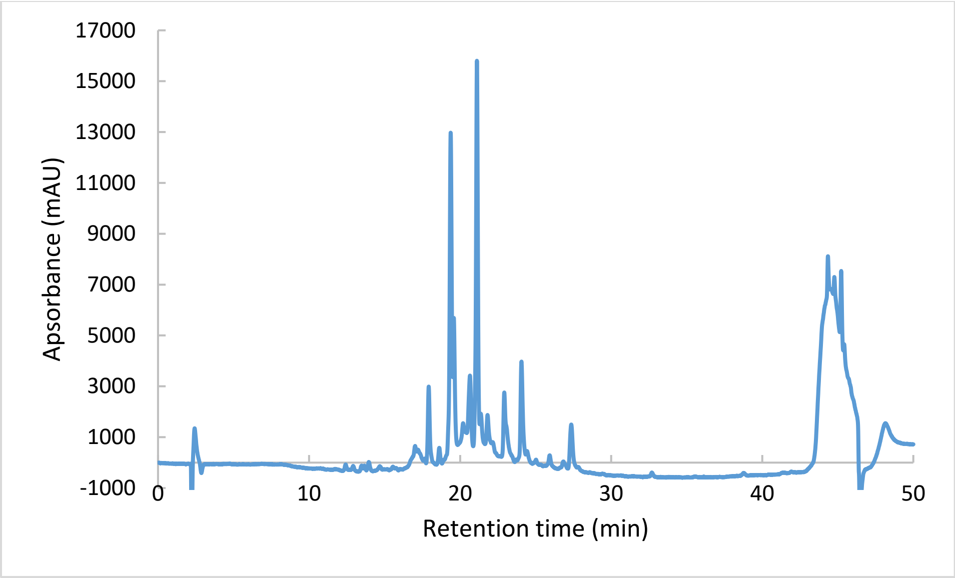

Figure S3: Chromatogram of E at 360 nm

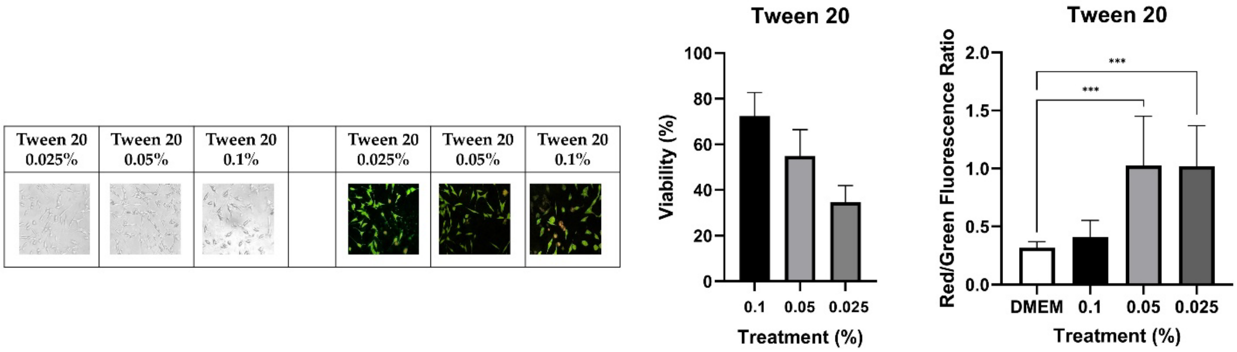

Figure S4. Positive control in cytocompatibility of Grape-Based Hydrogels Extracts on MRC-5 Cells: A) Bright-field and live/dead fluorescence microscopy images of MRC-5 fibroblasts after 24 h exposure to different concentrations of Tween 20, DMEM was used as the untreated control. B) MTT assay showing cell viability expressed as a percentage relative to the control. C) Red/green fluorescence ratio after EO/AB staining as an indicator of cell membrane integrity. Data are presented as M ± SD. Normality and homogeneity of variances were confirmed using the Shapiro–Wilk and Levene's tests, respectively, prior to ANOVA. Significance: $p < 0.05$ (*), $p < 0.01$ (**), $p < 0.001$ (***).

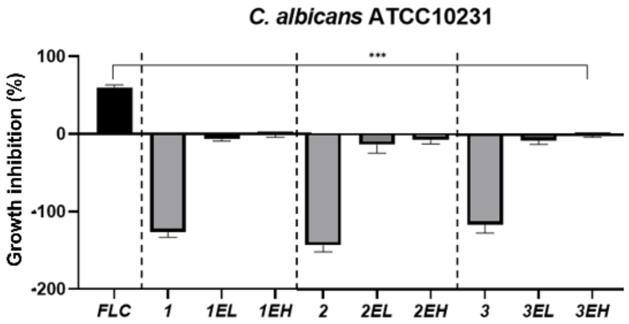

Figure S5. Antimicrobial Effects of Grape-Based Hydrogels Against *C. albicans* ATCC 10231 in Direct Contact Assay: Bacterial growth inhibition (%) after direct hydrogel contact determined by measuring OD_{600}. FLC = fluconazole (control). Hydrogels base (1, 2, 3), extract-enriched formulations with lower and higher concentration of grape skin extract (EL and EH respectively). Data are presented as M ± SD. Normality and homogeneity of variances were confirmed using the Shapiro–Wilk and Levene's tests, respectively, prior to ANOVA. Significance: $p < 0.05$ (*), $p < 0.01$ (**), $p < 0.001$ (***).

Table S1. Phenolic acid and flavonoid content (mg×10^{-2}/g ± SD) in grape sample.

Sample / Compound	E
Gallic acid	566 ± 6
3,4-DHB	N/A
3,5-DHB	N/A
Catechin	N/A
Chorogenic acid	302 ± 3
Caffeic acid	1051 ± 26
Syringic acid	119 ± 1
Epicatechin	5415 ± 39
p-Coumaric acid	126 ± 5
Ferulic acid	142 ± 1
Sinapic acid	473 ± 3
Rutin	330 ± 3
Naringin	3828 ± 54
Myricetin	908 ± 12
Morin	458 ± 14

Quercetin	232 ± 9
Naringenin	104 ± 11
Apigenin	707 ± 5
Crysin	77 ± 1

*SD – standard deviation, N/A – not available, DHB – Dihydroxy benzoic acid; E- grape skin extract.
